# Association between phase angle and daily creatinine excretion changes in critically ill patients: an approach to muscle mass

**DOI:** 10.3389/fphys.2024.1508709

**Published:** 2025-01-07

**Authors:** Patricio Vargas-Errázuriz, Natalia Dreyse, René López, Marcelo Cano-Cappellacci, Jerónimo Graf, Julia Guerrero

**Affiliations:** ^1^ Grupo Intensivo, Instituto de Ciencias e Innovación en Medicina (ICIM), Facultad de Medicina, Clínica Alemana Universidad del Desarrollo, Santiago, Chile; ^2^ Departamento de Paciente Crítico, Clínica Alemana de Santiago, Santiago, Chile; ^3^ Departamento de Farmacia, Clínica Alemana de Santiago, Santiago, Chile; ^4^ Physical Exercise Sciences Laboratory, Physical Therapy Department, University of Chile, Santiago, Chile; ^5^ Disciplinary Program of Physiology and Biophysics, Institute of Biomedical Sciences, Medicine Faculty, University of Chile, Santiago, Chile

**Keywords:** phase angle, daily creatinine excretion, critical care, muscle mass, bioelectrical impedance analysis

## Abstract

Assessing muscle mass in critically ill patients remains challenging. This retrospective cohort study explores the potential of phase angle (PA°) derived from bioelectrical impedance analysis (BIA) as a surrogate marker for muscle mass monitoring by associating it with daily creatinine excretion (DCE), a structural and metabolic muscle mass marker. In 20 ICU patients, we observed a linear relationship between PA° and DCE at initial (S1) and follow-up (S2) points, with Rho values of 0.78 and 0.65, respectively, as well as between their percentage changes (Rho = 0.80). Multivariate analysis confirmed a strong association between changes in PA° and DCE (adjusted R^2^ of 0.73), while changes in the extracellular water to total body water (ECW/TBW) ratio showed no significant association. This study establishes a relationship between a BIA-derived independent-weight parameter and DCE, highlighting the potential of PA° for muscle mass monitoring during acute changes, such as those seen in ICU settings. Integrating PA° into clinical practice could provide a non-invasive and reliable tool to enhance muscle assessment and support targeted interventions in critically ill patients.

## Introduction

Creatinine, a metabolic by-product of the creatine–phosphocreatine system, is synthesized in muscles and subsequently filtered and excreted by the kidneys (Bloch and Schoenheimer, 1941; Bonilla et al., 2021; Wyss and Kaddurah-Daouk, 2000). Creatinine reflects the activity of muscle metabolism, which is crucial for maintaining both muscle structure and function (Afting et al., 1981; Bonilla et al., 2021; Borsook and Dubnoff, 1947). Its production is intrinsically linked to high-energy-demand processes, such as muscle contraction and the maintenance of cell membrane potential (Bonilla et al., 2021; Wallimann et al., 2011).

In clinical practice, daily urinary creatinine excretion (DCE) is a reliable measure of whole-body muscle mass, showing a strong correlation under steady-state conditions (Forbes and Bruining, 1976; Heymsfield et al., 1983; Hoberman et al., 1948; Wang et al., 1996). This makes DCE a practical tool for assessing muscular changes (Cruz-Jentoft et al., 2019; Proctor et al., 1999; Reeds et al., 1978). Studies on age-related sarcopenia suggest that tracking changes in DCE over time provides a more accurate assessment of muscle loss than gold-standard methods (Proctor et al., 1999).

In critical care, wasting syndrome is a severe complication marked by early and sustained loss of muscle mass and strength. This condition is strongly associated with poorer outcomes, including impaired functional recovery and lower survival rates (Chen and Huang, 2024; Cheung et al., 2021; Fan et al., 2014; Schefold et al., 2020). Notably, DCE measured after ICU admission has been identified as a robust independent predictor of both short- and long-term mortality (Hessels et al., 2018; Schetz et al., 2014; Volbeda et al., 2024).

Measuring muscle mass in critically ill patients is challenging (Fazzini et al., 2023). Imaging techniques such as computed tomography (CT), magnetic resonance imaging (MRI), and dual-energy X-ray absorptiometry (DXA) are considered gold standards, but they are limited by logistical constraints and high costs (Looijaard et al., 2018). Ultrasound, although more feasible, is highly dependent on the operator and the methodology used (Fazzini et al., 2023). These limitations underscore the need for non-invasive, reliable tools to monitor muscle mass in the critically ill (Looijaard et al., 2018).

Bioimpedance analysis (BIA) is a non-invasive method for assessing muscle mass using whole-body electrical conductivity; its parameters strongly correlate with skeletal muscle mass measured by MRI in healthy individuals and have shown predictive value in monitoring malnutrition and sarcopenia in different populations. However, it is influenced by fluid shifts and metabolic alterations, complicating their interpretation (Bosy-Westphal et al., 2017; Janssen et al., 2000; Khalil et al., 2014; Kim et al., 2019; Lukaski et al., 2017; Moonen and Van Zanten, 2021).

Phase angle (PA°), a BIA-derived parameter, reflects the balance between resistance (R) and reactance (Xc), representing the resistive and capacitive properties of tissues (Barnett and Bagno, 1935; Ward and Brantlov, 2023). PA° is influenced by cell membrane integrity and the distribution of water between intracellular and extracellular compartments (Barnett and Bagno, 1935; Cimmino et al., 2023; Nescolarde et al., 2023; Ward and Brantlov, 2023). Healthy muscle tissue contributes significantly to reactance due to its higher cell membrane capacitance, which is associated with larger muscle fiber surface area and contractile properties (Yamada et al., 2016; Yamada et al., 2022). As muscle mass decreases, intracellular water shifts to the extracellular compartment, reducing reactance and, consequently, PA° (Akamatsu et al., 2022). Therefore, PA° reflects key physiological properties of the skeletal muscle, such as cell membrane integrity and hydration status, making it a plausible surrogate marker for assessing both muscle quality and quantity (Akamatsu et al., 2022; Baumgartner et al., 1988; Costa Pereira et al., 2024; Lukaski et al., 2019).

PA° has emerged as a reliable clinical marker associated with muscle mass, hydration, nutritional status, strength, quality of life, and mortality across diverse populations (Arab et al., 2021; Costa Pereira et al., 2024; Di Vincenzo et al., 2021; Garlini et al., 2019; Jiang et al., 2022; Lukaski et al., 2017; Nescolarde et al., 2023; Otsuka et al., 2022; Sardinha and Rosa, 2023; Yamada et al., 2016). Its prognostic value is well-established in various clinical settings, including intensive care units (ICUs), where PA° has been shown to predict mortality, prolonged ICU stays, and impaired functional recovery (Denneman et al., 2020; Ellegård et al., 2018; Garlini et al., 2019; Lima et al., 2022; Stapel et al., 2018; Stellingwerf et al., 2022; Thibault et al., 2016). Evidence indicates that PA° identifies patients with reduced muscle mass as measured by CT, underscoring its potential as a tool for assessing muscle loss in ICU (Looijaard et al., 2018; Looijaard et al., 2020). Furthermore, longitudinal changes in PA° have been recognized as predictors of functional decline in patients with septic shock, reinforcing its importance in critical care (Deana et al., 2024; Ellegård et al., 2018).

Despite these promising findings, critical gaps remain. In particular, the association between PA° and biomarkers of muscle metabolism, such as DCE, has not yet been explored. This study aims to examine the relationship between concurrent changes in PA° and DCE among critically ill patients with prolonged ICU stays. We hypothesize that PA° could serve as a reliable surrogate marker for DCE, providing valuable insights for monitoring muscle mass in critical care settings.

## Material and methods

This retrospective, single-center, observational cohort study utilized data from the RUCI Project, a single-center, non-experimental registry of critically ill ICU patients which collects anonymized demographic and clinical data from electronic medical records for observational studies and internal quality indicators. The study was approved by the institutional ethical committee (IRB 2012-53), and informed consent was waived due to its observational nature.

We included adult patients (>18 years) with ICU stays exceeding 4 days, a criterion chosen to ensure the inclusion of patients with significant clinical exposure to critical illness and excluding those with short ICU stays (Wollersheim et al., 2014). Eligible patients had concurrent DCE and PA° measurements obtained within the same 24-hour period on two separate occasions during their ICU stay. Only DCE measurements taken in steady state, defined by a serum creatinine variability of less than 0.3 mg/dL over the 24-hour urine collection period, were considered (Kellum et al., 2013). All consecutive patients from August 2022 to May 2024 were evaluated for inclusion.

### Variables

Patient characteristics such as age, gender, total body weight, body height, CrCl, SCr, and admission diagnosis were obtained from medical records. For severity assessment, Acute Physiology and Chronic Health Evaluation II (APACHE II) and Sequential Organ Failure Assessment (SOFA) scores were calculated. Serum creatinine (SCr) and urine creatinine were determined using the Jaffé compensated method without deproteinization (Roche, Cobas) (Table 1).

**TABLE 1:** Patient characteristics.

	(n = 20)
Male gender, n (%)	14 (70)
Age (years), median (range)	67.5 (61.3–73.3)
SOFA at ICU admission, median (range)	6 (4.8–10)
APACHE II, median (range)	19.0 (15.5–23.8)
Body mass index (kg/m^2^), median (range)	27.7 (23.8–33.3)
Admission ICU diagnosis:	
Respiratory failure, n (%)	14 (70)
Severe pneumonia, n (%)	14 (70)
Sepsis, n (%)	16 (80)
Emergency surgery, n (%)	4 (20)
Cardiovascular, n (%)	1 (5)
Other Variables:	
Vasopressors, n (%)	20 (100)
Mechanical ventilation, n (%)	20 (100)
ECLS, n (%)	2 (10)
Prone position, n (%)	14 (70)
Acute kidney injury, n (%)	16 (80)
ARC, n (%)	2 (10)
RRT during ICU stay, n (%)	7 (35)
Tracheostomy, n (%)	15 (75)
Immune status:	
Immunocompetent, n (%)	7 (35)
Hematooncologic, n (%)	4 (20)
Solid tumors, n (%)	1 (5)
Immunocompromised (non-cancer)	8 (40)

SOFA, sequential organ failure assessment score; APACHE II, acute physiology and chronic health disease classification system II; ECLS, extra corporeal life support; ARC, augmented renal clearance; RRT, renal replacement therapy.

We routinely collected 24-hour urine samples as part of standard ICU care to determine CrCl (24 h-CrCl) and nitrogen balance, in addition to performing body composition measurements with BIA in ICU patients requiring drugs and nutrition adjustments.

### BIA

The BIA was performed using the mBCA 525 portable bioelectrical analysis medical instrument (Seca, Germany); it comprises an electronic unit with a wireless touchscreen monitor linked to a measuring mat connected by cable to eight adhesive gel electrodes placed at defined anatomical sites, according to the manufacturer’s instructions. This equipment was chosen because it allows measurements to be performed with patients in bed without requiring any effort from them. Impedance was measured with a current of 100 uA at frequencies between 1 and 500 kHz. The duration of each BIA measurement was 75 s. PA° was automatically calculated by the mBCA 525 from the arctangent of the ratio of reactance to resistance using raw data at 50 kHz (Bosy-Westphal et al., 2006). Hydration status was evaluated by the extra-cellular water to total body water ratio (ECW/TBW) (Chung and Kim, 2021; Park et al., 2020; Rodríguez-Moguel et al., 2024).

### DCE

DCE was assessed through 24-hour urine collection via a catheter. To ensure the validity of the 24-hour collected urine samples, we double-checked the total urine volume recorded in the nurse clinical registry (hourly registration) and the laboratory report of each sample. As previously mentioned, only DCE measurements considered to be in steady state were included, defined by a SCr variability of less than 0.3 mg/dL over the 24-hour urine collection period.

### Changes in PA° and DCE

DCE and PA° measurements taken within the same 24-hour period on two separate occasions were designated as sets 1 (S1) and 2 (S2). The time intervals from ICU admission to S1 and from S1 to S2 were recorded in weeks. For analysis, differences between S1 and S2 were represented as Delta PA° and Delta DCE, which were expressed as percentage changes (Delta%PA° and Delta%DCE). Patients were categorized into two groups: Group 1 included those with a negative Delta%DCE, indicating a reduction in DCE between measurements, while Group 2 consisted of those with a positive Delta%DCE, reflecting an increase in DCE.

### Statistical methods

Statistical analysis was conducted using IBM SPSS 26 statistical software (IBM Corp, Armok, NY), while graphics were made with GraphPad Prism (Version 8). Continuous variables were expressed as medians (interquartile range—IQR) and categorical variables as frequency and percentages. Paired continuous measurements were analyzed using the Wilcoxon test. Spearman´s rank correlation coefficient was used to study the correlation between variables. The normality of distribution was evaluated by the Shapiro–Wilk test. A *p*-value of less than 0.05 was considered statistically significant. To explore the potential effect of the ECWTBW ratio on the relationship between concurrent changes in PA° and DCE, a linear regression model was performed. Delta%DCE was defined as the dependent variable, while Delta%PA and Delta%ECW/TBW were included as independent variables. Standard metrics were reported.

## Results

Of the study’s 20 patients, 14 (70%) were men, with a median age of 67.5 (61.3–73.3) years, height 173 (170–178) cm, weight 81 (71–98) kg, APACHE II score of 19.0 (15.5–23.8), and SOFA score of 6 (4.8–10).

The main admission diagnoses were sepsis and acute respiratory failure, reported in 16 (80%) and 14 (70%) patients, respectively. Regarding supportive interventions, all patients received vasopressors, mechanical ventilation, and sedation. Acute kidney injury was observed in 80% of patients, with 35% requiring renal replacement therapy. Additionally, six (30%) patients had active cancer under treatment, and 13 (65%) were immunocompromised (Table 1). The time elapsed between admission and S1 was 2.14 (0.57–4.71) weeks, while the interval between S1 and S2 was 3.14 (1.29–5.29) weeks.

**FIGURE 1:**
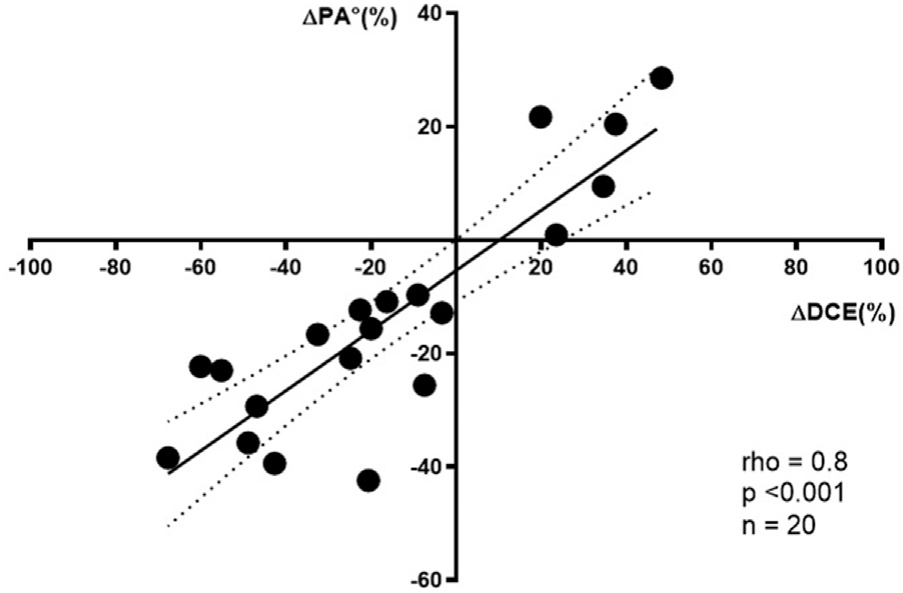
Correlation between changes in daily creatinine excretion (ΔDCE%) and phase angle (ΔPA%) in critically ill patients (n = 20).

Between S1 and S2, both DCE and PA° showed significant decreases (*p* = 0.025 and *p* = 0.007, respectively). The percentage changes in DCE and PA° were −20.3% (−45.8 to 14.0) and −16.1% (−28.4 to −1.7), respectively (Table 2). Significant correlations were observed between DCE and PA° at both time points (S1: Rho = 0.78; S2: Rho = 0.65; *p* < 0.001) and between their percentage changes (Rho = 0.80; *p* < 0.001) (Figure 1).

**TABLE 2:** Comparison of DCE, PA°, and ECW/TBW values at two time points (S1 and S2) in critically ill patients, including percentage changes.

		S1	S2	p	Delta %
Total	DCE (mg/24 h)	918 (651–1.350)	685 (529–858)	0.025	−20.3 (−45.8 to 14.0)
	PA (°)	3.9 (3.1–4.5)	3.1 (2.6–3.4)	0.007	−16.1 (−28.4 to −1.7)
	ECW/TBW (%)	47.8 (45.4–49.2)	48.7 (47.7–51.6)	0.002	3.8 (−6.0 to 6.0)
Group 1	DCE (mg/24 h)	1.004 (700–1.406)	655 (519–793)	0.001	−24.8 (−48.9 to −16.3)
n = 15	PA (°)	4.0 (3.4–4.8)	3.0 (2.6–3.4)	0.001	−22.3 (−35.8 to −12.8)
	ECW/TBW (%)	47.0 (44.7–48.8)	49.0 (47.7–51.6)	0.001	5.3 (3.0–7.3)
Group 2	DCE (mg/24 h)	645 (355–1.137)	830 (514–1.394)	0.043	34.6 (21.7–42.9)
n = 5	PA (°)	3.0 (2.3–3.7)	3.2 (2.8–4.3)	0.043	20.5 (5.2–25.2)
	ECW/TBW (%)	48.5 (47.4–53.2)	48.3 (46.1–52.6)	0.043	−0.8 (−3.0 to −0.4)

Values are presented as medians (interquartile range). Delta % represents the percentage change between S1 and S2 for each variable. Patients were categorized into two groups based on whether Delta% DCE was positive (Group 2) or negative (Group 1). *p*-values were calculated using the Wilcoxon signed-rank test to compare values between time points. ECW/TBW: extracellular water to total body water ratio; PA°: phase angle; DCE: daily creatinine excretion.

### Hydration status

The ECW/TBW ratio increased significantly from 47.8% (45.4%–49.2%) in S1 to 48.7% (47.7%–51.6%) in S2 (*p* = 0.002). The percentage change (Delta%ECW/TBW) was 3.8% (−6%–0%). A significant negative correlation was observed between the ECW/TBW ratio and both DCE and PA° at each time point (Table 3).

**TABLE 3:** Correlation matrix between DCE, PA°, ECW/TBW, and their percentage changes.

Variables	Rho	*p*-value
DCE - PA° (S1)	0.78	<0.001
DCE - PA° (S2)	0.65	<0.001
Delta%DCE - Delta%PA°	0.80	<0.001
DCE - ECW/TBW (S1)	−0.73	<0.001
DCE - ECW/TBW (S2)	−0.81	<0.001
Delta%DCE - Delta%ECW/TBW	−0.72	<0.001
PA° - ECW/TBW (S1)	−0.88	<0.001
PA° - ECW/TBW (S2)	−0.80	<0.001
Delta%PA° - Delta%ECW/TBW	−0.86	<0.001

Spearman’s rank correlation coefficients (Rho) and *p*-values are reported for the relationships between DCE, PA°, ECW/TBW, and their percentage changes at two time points (S1 and S2). Delta values represent the percentage change between S1 and S2 for each variable.

In the multivariate analysis, Delta%PA° and Delta%ECWTBW were included as variables to assess their association with Delta%DCE. The model was statistically significant (*p* < 0.001), with an adjusted R^2^ of 0.726. A significant association was identified between Delta%PA° and Delta%DCE (*p* < 0.001), while no significant relationship was found between Delta%ECWTBW and Delta%DCE (*p* = 0.965) (Table 4). In the residual analysis, the Shapiro–Wilk test confirmed the normality of residuals. The Durbin–Watson statistic was 2.465, indicating no significant autocorrelation.

**TABLE 4:** Multivariate analysis.

Variable	B	SE	p	95% CI lower	95% CI upper
Constant	3.88	5.43	0.48	−7.6	15.33
Delta%PA	1.41	0.39	0.002	0.6	2.2
Delta%ECW/TBW	−0.09	1.99	0.965	−4.3	4.12

B, unstandardized coefficient; SE, standard error; p, *p*-value; CI, confidence interval. Results are from a multivariate linear regression model evaluating associations between Delta%DCE (dependent variable) and Delta%PA° and Delta%ECW/TBW (independent variables). The model was statistically significant (*p* < 0.001), with an adjusted R^2^ of 0.726.

### Subgroup analysis

Based on Delta%DCE, patients were categorized into two groups: those with a negative change (Group 1) and those with a positive change (Group 2). Complete agreement was observed between directional changes in DCE and PA° in all patients.

**FIGURE 2:**
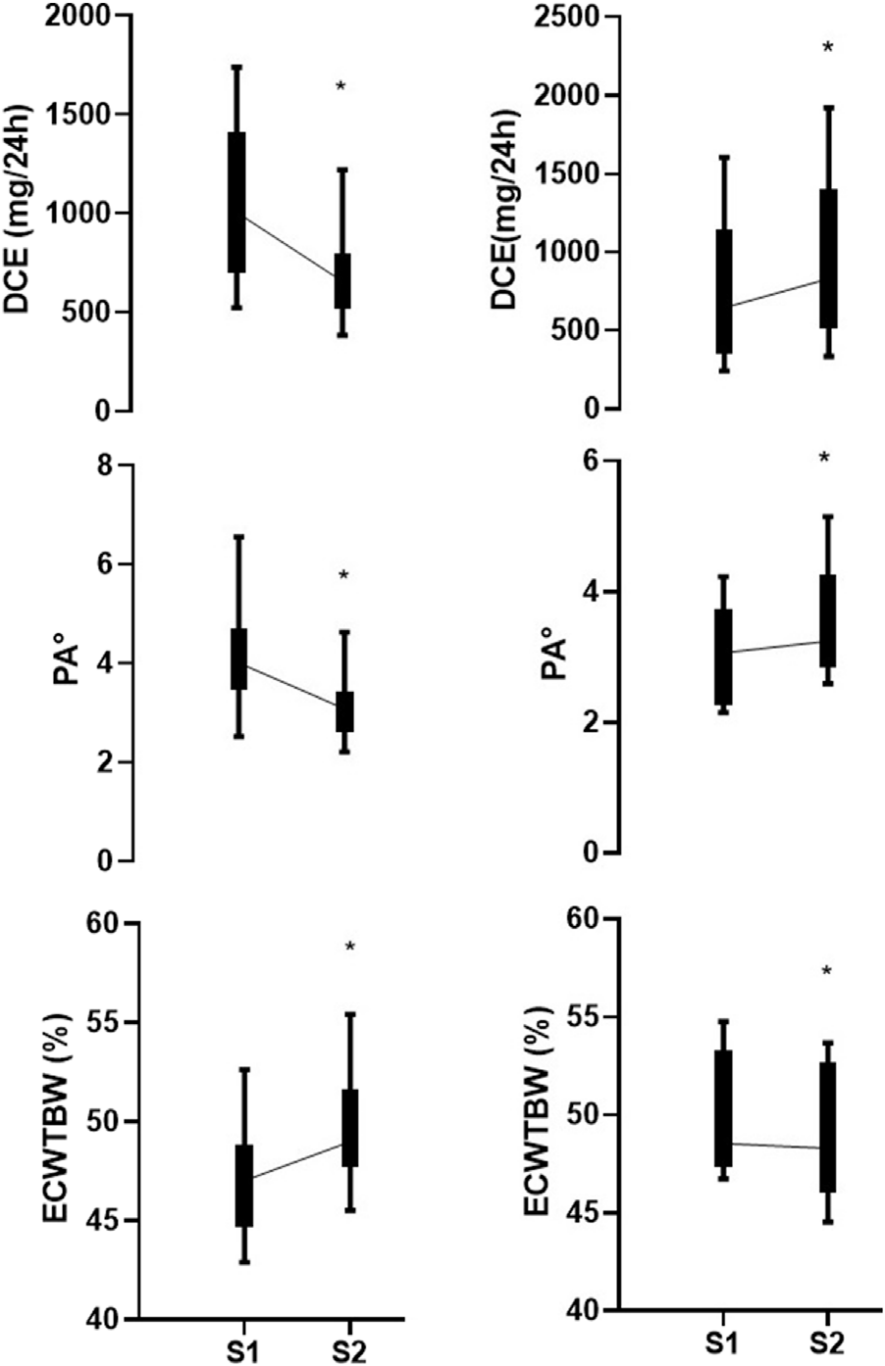
Comparison of changes in daily creatinine excretion (DCE), phase angle (PA°), and extracellular water to total body water ratio (ECW/TBW) between set 1 (S1) and set 2 (S2) in critically ill patients. Left panels represent patients with negative ∆DCE, indicating a decrease in DCE between S1 and S2, while right panels show those with positive ∆DCE. Median values with interquartile ranges are shown for DCE (mg/24 h), PA°, and ECW/TBW (%). *p < 0.05.

In Group 1 (n = 15), both DCE and PA° showed significant decreases between S1 and S2 (*p* = 0.001 for both), accompanied by a significant increase in the ECW/TBW ratio (*p* = 0.001). In contrast, Group 2 (n = 5) demonstrated significant increases in both DCE and PA° (*p* = 0.043 for both), along with a significant decrease in the ECW/TBW ratio (*p* = 0.043) (Table 2; Figure 2).

## Discussion

Our study revealed significant correlations between PA° and DCE at two different time points (Rho = 0.78 for S1 and Rho = 0.65 for S2), as well as between their percentage changes (Rho = 0.80). To our knowledge, this is the first study to investigate the correlation between DCE and a weight-independent muscle marker obtained by BIA in ICU patients, yielding results comparable to those reported in a physiological aging study under ideal conditions (Proctor et al., 1999). In that study, a strong correlation (r = 0.81) was observed between DCE and total muscle mass measured by DXA. Similarly, Bosy-Westphal et al. (2006) showed that BIA-derived parameters correlate with DXA measurements in healthy individuals. While these findings validate both DCE and BIA against reference techniques, their relationship had not been explored in the ICU setting, where altered body-water balance and significant muscle mass loss could affect results (Denneman et al., 2020). Nonetheless, our findings support the validity of using BIA-derived parameters or DCE to assess muscle mass in this context.

The relationship between PA° and DCE is not perfectly linear, comparable to the correlation between DCE and DXA (Proctor et al., 1999). This discrepancy can be partly explained by the magnitude and proportional distribution of total body water—variables that this technique does not completely differentiate from metabolically active muscle mass (Proctor et al., 1999). Similarly, changes in PA° obtained by BIA also partially reflect changes in tissue hydration status (Denneman et al., 2020). In our sample, we observed an increase in ECW/TBW between S1 and S2, as is well described in critical ICU patients (Chae et al., 2021), and we found a high negative correlation between the ECW/TBW ratio and DCE in S1 (Rho = −0.73), which increased further in S2 (Rho = −0.81). Conversely, the correlation coefficient between PA° and DCE decreased from 0.73 in S1 to 0.65 in S2. These results could be explained by changes in ECW/TBW, as hydration status directly influences the precision of BIA-derived variables, consistent with Chae et al. (2021), Kim et al. (2019), and Nakanishi et al. (2019).

To further explore this relationship, our multivariate analysis supports a strong relationship between changes in PA° and DCE (adjusted R^2^ = 0.73), with Delta%ECW/TBW having a limited effect on the model under the studied clinical conditions. This finding aligns with that of a previous study that utilized CT and BIA measurements in older adults, where BIA was not influenced by water distribution (Yamada et al., 2014). Nevertheless, these results should be regarded as exploratory, highlighting useful trends that require further validation in larger cohorts. This suggests that future predictive models of DCE based on BIA could incorporate changes in ECW/TBW to enhance accuracy and reliability.

Notably, we found complete agreement between directional changes in DCE and PA° among those who gained, lost, or maintained their muscle mass. This finding underscores a consistent and strong correlation between DCE and PA°, suggesting that both variables may reflect interconnected physiological processes. Furthermore, the absence of discrepancies in trends across different scenarios (muscle gain, loss, or maintenance) highlights the potential of PA° as a reliable marker for tracking changes in DCE. This finding could have significant clinical implications that support the use of PA° as a tool for evaluating the metabolic dynamics of muscle in these patients over time.

Remarkably, both PA° and DCE exhibited significant declines in a considerable number of patients, with DCE decreasing by almost 25% in 15 (75%) patients over a median period of 4 weeks (IQR 3–5). This decline aligns with previously reported rates in the literature (0.5%–1% reduction per day in the ICU) (Deana et al., 2024; Fazzini et al., 2023; Schetz et al., 2014; Volbeda et al., 2021). Additionally, it is noteworthy that this group showed a significant increase in the ECW/TBW ratio (3.0%–7.3%), likely due to the greater severity of their condition (Chae et al., 2021). These changes likely reflect the pronounced muscle mass loss attributable to the high severity of the patients included in this study (Chen and Huang, 2024; Fazzini et al., 2023).

Interestingly, in five patients both DCE and PA° increased while the ECW/TBW ratio significantly decreased. At the time of this study, they were participating in active motor rehabilitation, which was not interrupted, potentially explaining these results. Our findings highlight the potential of PA° measures not only for early risk stratification but also to document recovery in the later phases of critical illness, particularly in chronic critically ill patients. These results suggest that the structural and functional impairments commonly observed in ICU survivors may be reversible. With appropriate rehabilitation, recovery can be objectively measured and tracked. However, due to the small sample size, no definitive conclusions can be drawn. Future studies could focus on the assessment of PA° in patients recovering from critical illness and evaluate its association with various motor, respiratory, and functional scales during recovery, along with serial measurements of muscle ultrasound.

The observed correlation between concurrent PA° and DCE changes likely reflects the dependence on underlying mechanisms involving muscle intracellular capacitance and muscular metabolic activity (Yamada et al., 2016). Among the multiple mechanisms associated with wasting syndrome are protein imbalance, mitochondrial dysfunction, altered sarcoplasmic reticulum structure and function, neuropathy, and notably, abnormal electrical excitability (Chen and Huang, 2024). These mechanisms can be related to both changes in cellular capacitance and the metabolic activity of the creatine–phosphocreatine system, particularly in cellular functions with high energy demands, such as muscle contraction and the maintenance of cell membrane potential (Bonilla et al., 2021; Wallimann et al., 2011).

Unlike other muscle mass variables obtained by BIA, such as body cell mass (BCM), skeletal muscle mass (SMM), or the fat-free mass index (FFMI), PA° is not dependent on precise total body weight estimations (Cimmino et al., 2023; Denneman et al., 2020; Kim et al., 2019; Nakanishi et al., 2019). PA° is derived from raw data from BIA evaluation on resistance and reactance (Ward and Brantlov, 2023). This characteristic is particularly advantageous in ICU settings, where obtaining accurate total body weight measurements can be challenging due to patient severity and a high frequency of measurement issues and errors (Bloomfield et al., 2006; Moonen and Van Zanten, 2021). Total body weight interpretations in ICU patients are also complicated by dynamic changes in total body water and the proportion between extracellular and intracellular compartments (Chae et al., 2021; Denneman et al., 2020).

Another strength of the PA° is that it does not require the use of formulas typically included in BIA devices, which provide a “volumetric” estimation of muscle mass (usually in kg) derived from mathematical extrapolations validated in healthy populations, including athletes (Moonen and Van Zanten, 2021). Therefore, PA° may serve as a more reliable and objective tool for interpreting body composition, particularly in the context of longitudinal muscle monitoring (Ellegård et al., 2018; Looijaard et al., 2020; Moonen and Van Zanten, 2021).

In our study, 65% of patients were either undergoing active cancer treatment or were under immunocompromised conditions closely associated with sarcopenia and frequently complicated by acute kidney injury (AKI), the most common organ dysfunction in this population. Both AKI and sarcopenia limit the administration of curative therapies and can also arise as adverse effects of anticancer treatments, thus significantly contributing to poor clinical outcomes (Anjanappa et al., 2020; Darmon et al., 2015; Rogeri et al., 2020; Schefold et al., 2020). Evidence indicates that AKI reduces the curative rate of therapy and increases mortality, underscoring the need for accurate renal function evaluation in this high-risk group (Rosner and Perazella, 2019). Although validated tools for CrCl estimation in sarcopenic and critical patients are lacking (Kadivarian et al., 2022; Nankivell et al., 2020; Schetz et al., 2014; Sunder et al., 2014; Volbeda et al., 2021), personalized assessments of muscle mass could improve CrCl predictions (de Jong et al., 2019; Donadio, 2017; Donadio et al., 2017; Nankivell et al., 2020; Rule et al., 2009). PA° could serve as an individualized surrogate for DCE in CrCl for more accurate predictions. Our study team is currently developing a predictive function based on PA° to estimate CrCl.

In ICUs, DCE is no longer used exclusively for evaluating muscle mass and for CrCl assessment; issues limit its use, likely due to the extended time required for urine collection (24 h) and precision concerns, such as the need for a urinary catheter and team coordination for more accurate urine collection.

Another important consideration for correctly interpreting DCE is the stability of the glomerular filtration rate (GFR). Acute changes in GFR can lead to errors in DCE measurements (Kassirer, 1971). One tool for evaluating stability is monitoring SCr, but there are no established definitions for acceptable variations. Our criterion of accepting an SCr variability of less than 0.3 mg/dL over 24–48 h was based in KDIGO definition (Kellum et al., 2013). Since GFR stability is uncommon in critically ill patients, we included only those patients with two consecutive DCE measurements that meet our stability criteria. This requirement explains the limited sample size in this cohort.

The clinical significance of these findings lies in demonstrating, for the first time, the strong association between bioelectrical and metabolic changes, using two variables previously recognized for their prognostic value in critically ill patients (Hessels et al., 2018; Lima et al., 2022; Stellingwerf et al., 2022). This suggests that PA° could transcend its role as just a prognostic marker, potentially providing biologically relevant information beyond traditional assessments of muscle thickness or density (Yamada et al., 2016). Moreover, PA° could serve as a valuable tool for longitudinal follow-up, offering insights into the progression and dynamics of wasting syndrome, even during the recovery phase following ICU discharge.

In addition, its simplicity and practicality enable widespread adoption, allowing it to be used as a frequent clinical measurement while providing valuable information to clinicians without requiring patient displacement, radiation exposure, patient effort or cooperation, invasive devices such as urinary catheters, or reliance on operator precision or expertise. These characteristics make it a promising tool for use in ICU settings, creating opportunities for future research to evaluate the effects of nutritional or physiotherapy interventions over time, as has already been demonstrated in sports medicine and aging (Otsuka et al., 2022; Sardinha and Rosa, 2023).

## Limitations and strengths

This retrospective, single-center study with a small sample size may lack external validity. Variability in measurement timing and the absence of additional muscle markers limit a comprehensive understanding of the utility of PA°, highlighting the need for further validation in larger cohorts.

## Strength

Despite these limitations, this study demonstrates a plausible physiological association in a highly selected cohort of critically ill patients. These patients had severe illnesses characterized by at least two organ dysfunctions, high requirements for invasive life support, and underlying conditions with significant clinical impact. These characteristics were reflected in extended ICU stays, making this cohort a representative subset of ICU patients who are particularly affected by muscle mass loss and potentially more likely to benefit from future interventions (Wozniak et al., 2024).

Another strength was the rigorous study design, which included only precise and prolonged measurements of DCE under strict renal function stability criteria, along with concurrent BIA measurements to minimize potential confounding variables. While this cohort was highly selected and while inclusion criteria were stringent to ensure reliable DCE data, it was specifically designed to demonstrate its association with PA°, a simple, non-invasive tool that does not require such restrictions for its use in clinical practice.

## Future research

Because BIA does not need measurement skills and can easily be used by any operator, it is clinically useful for developing tools which could be applied for muscle mass monitoring. Given its electrical nature, dependent on resistance and reactance, PA° has certain known weaknesses when assessing muscle mass, such as the effect of body water on resistance and conductor geometry on reactance (Kim et al., 2019; Nakanishi et al., 2019). Therefore, supplementing its analysis with variables such as ECW/TBW, height, or muscle thickness (obtained by ultrasound) may be a way of improving its accuracy compared to reference techniques such as CT (Kim et al., 2019).

Future research should focus on validating PA° as a surrogate marker for DCE in larger ICU populations. Prospective studies could explore the impact of interventions based on PA° monitoring on patient outcomes. Additionally, investigating the relationship between PA° and other markers of muscle mass and function could provide a more comprehensive understanding of its utility in critical care. Another promising line of investigation is the clinical use of PA° for predicting creatinine clearance (CrCl), thus potentially enhancing the accuracy of renal function assessment in critically ill patients.

## Conclusion

This study demonstrates a significant association between PA° and DCE changes in critically ill patients, suggesting that PA° could serve as a surrogate marker for DCE. This non-invasive, rapid assessment tool could offer valuable insights for sequentially monitoring muscle mass changes in critical care. Integrating PA° measurements into clinical practice could enhance patient monitoring and support a more individualized approach to care, enabling timely nutritional and therapeutic interventions to mitigate muscle loss and improve ICU outcomes.

## Data Availability

The raw data supporting the conclusions of this article will be made available by the authors, without undue reservation.
